# Accurate measurement of gene copy number for human alpha-defensin *DEFA1A3*

**DOI:** 10.1186/1471-2164-14-719

**Published:** 2013-10-20

**Authors:** Fayeza F Khan, Danielle Carpenter, Laura Mitchell, Omniah Mansouri, Holly A Black, Jess Tyson, John AL Armour

**Affiliations:** School of Biology, Queen’s Medical Centre, University of Nottingham, Nottingham, NG7 2UH UK

**Keywords:** CNV, Defensin, GWAS, *DEFA1A3*, PRT

## Abstract

**Background:**

Multi-allelic copy number variants include examples of extensive variation between individuals in the copy number of important genes, most notably genes involved in immune function. The definition of this variation, and analysis of its impact on function, has been hampered by the technical difficulty of large-scale but accurate typing of genomic copy number. The copy-variable alpha-defensin locus *DEFA1A3* on human chromosome 8 commonly varies between 4 and 10 copies per diploid genome, and presents considerable challenges for accurate high-throughput typing.

**Results:**

In this study, we developed two paralogue ratio tests and three allelic ratio measurements that, in combination, provide an accurate and scalable method for measurement of *DEFA1A3* gene number. We combined information from different measurements in a maximum-likelihood framework which suggests that most samples can be assigned to an integer copy number with high confidence, and applied it to typing 589 unrelated European DNA samples. Typing the members of three-generation pedigrees provided further reassurance that correct integer copy numbers had been assigned. Our results have allowed us to discover that the SNP rs4300027 is strongly associated with *DEFA1A3* gene copy number in European samples.

**Conclusions:**

We have developed an accurate and robust method for measurement of *DEFA1A3* copy number. Interrogation of rs4300027 and associated SNPs in Genome-Wide Association Study SNP data provides no evidence that alpha-defensin copy number is a strong risk factor for phenotypes such as Crohn’s disease, type I diabetes, HIV progression and multiple sclerosis.

**Electronic supplementary material:**

The online version of this article (doi:10.1186/1471-2164-14-719) contains supplementary material, which is available to authorized users.

## Background

The majority of human copy number variants (CNVs) are simple di-allelic polymorphisms, generally involving variable deletion of non-coding sequences. However, a small but interesting subgroup of CNVs displays multi-allelic polymorphism for the copy number of a gene or cluster of genes. Examples include polymorphism for the copy number of *CCL3L1* and *CCL4L1*[1–3], of *FCGR3A* and *FCGR3B*[4, 5], and of a cluster of human beta-defensin genes on chromosome 8 [6–8]. In all these cases, associations of gene copy number with important medical phenotypes have been reported – of *CCL3L1*/*CCL4L1* with HIV infection [2, 9–11], of *FCGR3B* with systemic autoimmune disorders [4, 5], and of beta-defensins with Crohn’s disease and psoriasis [12, 13]. In the case of Crohn's disease, the associations proposed with the beta-defensin CNV have attracted controversy, particularly related to the confidence with which CNV states can be called [14, 15].

Establishing robust evidence for these associations is made considerably more difficult by the technical challenge of determining accurate measures of copy number [16]. Although most severe when the copy numbers are high, as in the case of the beta-defensins (2-12 copies), accuracy of copy number measurement is still an important issue in the interpretation of association data even when gene copy numbers are relatively low, as in the case of *CCL3L1*/*CCL4L1* (0-4 copies in Europe) [17–22]. Typing copy number by real-time PCR may be subject to errors that compromise the accuracy of association studies [16]. These errors may arise from differences in the physicochemical state of DNA samples that alter the relative behaviour of test and reference loci [18, 23]. At high copy numbers the level of relative precision required to distinguish integer copy number states with accuracy may simply be beyond the capabilities of real-time PCR, however carefully it is performed [14, 19]. For example, measurement error of only about 10% in analysis of a sample with a true copy number of 6 would result in an incorrect integer call. The quality-control difficulty created by performing case–control association studies of multi-allelic CNVs is compounded by the observation that no simple SNP tags that can act as surrogates for the determination of gene copy number have been identified to date.

Alternative approaches have been explored for the determination of copy number at multi-allelic loci that are simultaneously convenient, economic and accurate. For some but not all such loci [16], MLPA appears to provide an appropriate level of accuracy to call most integers correctly. Approaches involving Paralogue Ratio Tests (PRTs), which determine the representation of a test locus relative to a co-amplified reference locus, have also been successful in determining accurate copy number measures for even some of the more challenging loci [24–26]. Side-by-side comparisons [14, 27] appear to suggest greater accuracy of PRT compared with real-time PCR for robust and reproducible determination of copy number at multi-allelic CNVs. In addition to PRTs, measurement of paralogous ratios for allelic variants (microsatellites or indels) between variable repeats within a sample have also been valuable in supplementing information on gene copy number. PRT measurements in combination with allelic variant ratios have previously been used successfully in multiplex measurement systems for *CCL3L1*/*CCL4L1*[26], *FCGR3A/B*[28] and beta-defensins [14, 29].

The cluster of human alpha-defensin genes on chromosome 8 includes the genes *DEFA1* and *DEFA3*, which are copy-variable [7, 30, 31]. The genes *DEFA1* and *DEFA3* differ only by a single base substitution in the coding sequence, corresponding to a single amino acid difference between the peptides encoded. These genes appear to be interchangeable occupants of a 19 kb copy-variable repeat unit, with both *DEFA1* and *DEFA3* gene number showing variation. For this reason, Aldred *et al.*[30] suggested the composite designation *DEFA1A3* for the copy-variable locus. The *DEFA1* and *DEFA3* genes lead (after proteolytic processing) to the expression of three distinct antimicrobial peptides, generally designated as HNP-1, -2, and -3. High levels of these peptides are found in the granules of neutrophils [32, 33], and a small-scale study has suggested that the expression level of the peptides is correlated with gene copy number [7].

Serious technical challenges are posed by the accurate measurement of the multi-allelic copy number variation displayed by *DEFA1A3*, because most individuals have 6 or more repeats. A full characterisation of the variation should also include a separate determination of gene copy numbers for *DEFA1* and *DEFA3*. Furthermore, the existence of one repeat per haplotype differing substantially in sequence from others (the “partial repeat” [30]) makes application of many standard methods problematic. These factors may underlie the failure to score this CNV in the WTCCC CNV study, which adopted very thorough and carefully controlled approaches to CNV typing [34].

In this study, we apply and combine a range of measurement methods to determine the copy number of *DEFA1A3*, and to define the relative contribution of the *DEFA1* and *DEFA3* gene variants. This work has allowed us to derive a consistent characterisation of copy number variation among 589 European samples. Our data allow us to identify a single SNP that effectively tags low, medium, and high-copy number states, which can therefore act as a convenient surrogate for approximate *DEFA1A3* gene copy number in high-throughput studies.

## Results

**Figure 1:**
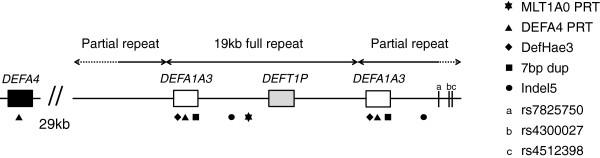
**Schematic map of the** ***DEFA1A3*** **CNV region, based on the human genome assembly (March 2006, NCBI36/hg18), and showing the expected structure of a 2-copy allele.** *DEFA1A3* genes are found in the copy-variable 19 kb full repeat sequence and (with exactly one copy per haplotype) in the centromeric partial repeat. The positions of the three SNPs rs7825750, rs4300027 and rs4512398 in the centromeric flanking DNA are indicated as a, b and c respectively. The specific measurement points used in this study in the determination of copy number are shown as differently-shaped symbols below the relevant locations, with the nearby *DEFA4* gene providing the reference locus for the DEFA4 PRT measurement (triangles). The MLT1A0 PRT (star) has a target site only in full, and not partial, repeat sequences, and for this reason measures one copy less per haplotype than the other assays; calibration of MLT1A0 PRT values is undertaken using the predicted total repeat unit count per sample, for ease of comparison with other measures.

The copy-variable alpha-defensin genes *DEFA1* and *DEFA3* are arranged in a variable tandem repeat on chromosome 8, in which each haplotype carries a single copy of a centromeric “partial” repeat and a variable number (including zero) of 19 kb “full” repeat units (Figure 1). Our measurements of copy number at *DEFA1A3* were based on five independent measures – two paralogue ratio tests (PRTs) and three measurements of ratios between variants within the variable repeat array. The ratio measurements included two examples of indels (one of 5 bp, the other of 7 bp), as well as the single-base substitution that distinguishes the variant genes *DEFA1* and *DEFA3* (Figure 1).

We applied these PRT measurements to evaluate *DEFA1A3* copy number in 600 unrelated DNA samples from Europeans (120 unrelated HapMap CEU phase 1 and 2 samples, and 480 samples from ECCAC HRC plates 1-5), calibrating the PRT ratios against samples of known copy number (see “Methods”). Of these 600 samples, 11 (1.83%) failed to produce adequate data (at least two measurements, including at least one PRT measurement), so that we obtained useful results for 589 unrelated European samples. Starting from “gold standard” DNA samples for which total *DEFA1A3* copy number had been inferred from restriction fragment lengths [30], we developed a secondary set of reference samples, drawn from publicly available sources; these were validated both using multiple measurements against the original reference samples as well as segregation within pedigrees (see below). These new reference samples are specified in the Methods, and listed separately in (Additional file 1: Table S2).

**Figure 2:**
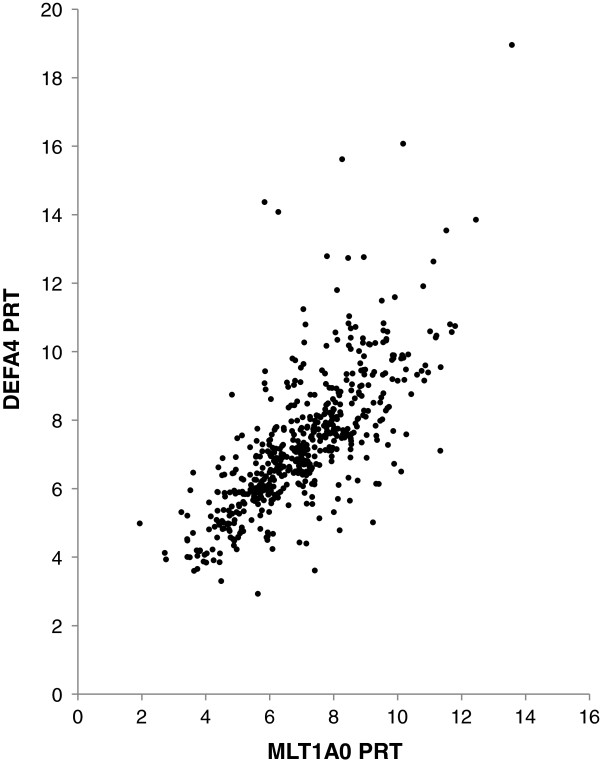
**Copy number estimates determined by MLT1A0 PRT1 plotted against the corresponding copy number measure from** ***DEFA4*** **PRT, showing the correlation between the two measures (r** ^**2**^ **= 0.731) and some evidence of clustering around integer values of 4, 5 and 6.**

The full set of results obtained is available as Additional file 2 (see also Additional file 1, section 3). The individual accuracy of each PRT method can be assessed by comparing the inferred unrounded copy number with the integer copy number deduced from overall analysis of each sample (see below) to give a normalised measurement – for example, a PRT measurement of 6.3 for a sample with a true copy number of 6 would have a normalised value of 1.05. The distributions of PRT measurements normalised relative to the (assumed) correct value are shown in (Additional file 1: Figure S1a and S1b). The two PRTs appear to exhibit similar levels of variation around the mean value, with standard deviations of normalised measurements of 0.114 and 0.12 for MLT1A0 and DEFA4 PRTs respectively. The distribution of normalised values conforms well to a Gaussian distribution over the central range of -2 < z < 2. However, normalised ratios from MLT1A0 PRT appear to have an excess of outliers at z values below about -2; see (Additional file 1: Figure S2a). The measures from the two PRT systems (MLT1A0 and *DEFA4*, Figure 2) correlate well with each other (r^2^ = 0.731), and although there is some indication of clustering around integer values for copy numbers of 4, 5 and 6, the clusters are less distinct at higher copy numbers.

In addition to the PRT measurements, three allelic ratio methods were developed. These are practically very useful measures in verifying copy number measurements because of their empirical accuracy, but can be limited in the information they provide; for example, a ratio of 2:1 is consistent with a copy number of 3, but also any multiple of 3. Examples of individual samples typed by these methods, with corresponding inferences about total copy number and variant constitution, are shown in Table 1.

**Table 1:** **Examples of integer copy number inference from PRT and ratio data**

Sample ID	MLCN	Minimum ratio (MR)	MLT1A0 PRT	DEFA4 PRT	indel5 ratio	indel5 integers	DefHae3 ratio	DefHae3 integers	7bpdup ratio	7bpdup integers
NA12751	7	511.47	7.07	7.03	1.32	4:3	2.38	5:2	0.41	2:5
C0052	8	152.11	8.30	7.70	1.07	4:4	5.66	7:1	0.60	3:5
NA12749	5	21.99	5.71	5.21	(No data)	-	3.70	4:1	3.74	4:1
C0157	9	10.50	9.09	9.02	1.18	5:4	6.12	8:1	0.78	4:5
NA12778	6	3.26	(No data)	7.59	1.12	3:3	5.49	5:1	0.21	1:5

Examples of copy number inference from PRT and ratio data. For each sample the table shows the MLCN (maximum likelihood copy number) derived by combining information from different measures (see text and Table 2) and the minimum ratio (MR) score indicating the confidence with which that copy number is assigned. For the three ratio tests (indel5, DefHae3 and 7bpdup), the measured ratio is shown alongside the integer interpretation derived from the MLCN that best fits the observed ratio. Samples are arranged in decreasing order of confidence, as reflected in decreasing values of the minimum ratio (MR).

Information was combined from different measurements using a likelihood-based approach similar to that employed by Aldhous *et al.* for beta-defensins [14]. Gaussian distributions were used to model the expected outcomes relative to the true copy number. The probabilities associated with each of the five measures (MLT1A0 PRT, *DEFA4* PRT, and ratios of alleles for indel5 (5 bp deletion), 7bpdup (7 bp duplication), and *DEFA1*:*DEFA3*) are combined by multiplication to derive an overall measure of relative likelihood for each copy number (Table 2). We refer to the copy number best supported by the combined data as the “Maximum Likelihood Copy Number” (MLCN), and the factor by which the support for that copy number exceeds the next best copy number (the “minimum ratio”, MR) is an index of confidence in the assignment of the copy number. Further details are given in the Additional file 1, Section 1. The output from this analysis for the 589 samples typed in this study is available as Additional file 3.

**Table 2:** **Examples of ML analysis, showing copy numbers in the range 2-10**

				Relative likelihood values for								
			Measured value	N = 2	N = 3	N = 4	N = 5	N = 6	N = 7	N = 8	N = 9	N = 10
Sample	NA12751	PRT1 (MLT1A0)	7.07	1.28E-139	2.84E-40	1.50E-11	6.82E-04	0.276	1	0.471	0.091	0.012
MLCN	7	PRT2 (DEFA4)	7.03	2.15E-137	1.97E-39	2.37E-07	0.010	0.458	1	0.544	0.156	0.034
Minimum ratio 511.47		indel5	1.32	0.044	2.02E-03	0.044	0.318	0.044	0.880	0.068	1	0.318
		DefHae3	2.38	5.67E-30	0.099	0.626	0.038	0.099	0.983	0.626	0.172	0.734
		7bpdup	2.43	6.06E-06	0.343	0.155	6.25E-03	0.343	1	0.155	0.343	0.970
		**Combined**		**4.87E-312**	**4.41E-83**	**1.76E-20**	**6.14E-10**	**2.20E-04**	**1**	**1.96E-03**	**9.67E-04**	**1.10E-04**
				**N = 2**	**N = 3**	**N = 4**	**N = 5**	**N = 6**	**N = 7**	**N = 8**	**N = 9**	**N = 10**
Sample	NA12749	PRT1 (MLT1A0)	5.71	6.56E-75	4.43E-18	5.99E-04	0.538	1	0.201	0.019	1.44E-03	1.16E-04
MLCN	5	PRT2 (DEFA4)	5.21	3.29E-56	3.55E-12	0.102	1	0.527	0.095	0.012	1.50E-03	2.00E-04
Minimum ratio 21.99		indel5	(-)	1	1	1	1	1	1	1	1	1
		DefHae3	3.70	2.95E-106	1.49E-12	0.045	1	0.366	0.057	0.045	0.550	1
		7bpdup	3.74	9.24E-114	4.16E-12	0.124	0.873	0.111	7.28E-03	0.124	0.863	0.873
		**Combined**		**0**	**2.08E-52**	**7.25E-07**	**1**	**0.045**	**1.68E-05**	**2.72E-06**	**2.19E-06**	**4.31E-08**
				**N = 2**	**N = 3**	**N = 4**	**N = 5**	**N = 6**	**N = 7**	**N = 8**	**N = 9**	**N = 10**
Sample	C0157	PRT1 (MLT1A0)	9.09	1.22E-272	1.34E-89	9.08E-31	2.19E-13	9.13E-06	0.022	0.478	1	0.612
MLCN	9	PRT2 (DEFA4)	9.02	3.54E-267	1.78E-87	3.73E-19	8.65E-09	5.47E-04	0.086	0.650	1	0.649
Minimum ratio 10.50		indel5	1.18	0.503	1.62E-04	0.503	0.058	0.503	0.329	0.503	0.637	0.503
		DefHae3	6.12	0	1.88E-63	6.26E-18	7.72E-06	0.053	0.734	0.958	0.486	0.171
		7bpdup	0.78	0.194	1.54E-05	0.194	0.351	0.194	0.938	0.194	0.985	0.351
		**Combined**		**0**	**3.66E-247**	**6.79E-67**	**9.71E-28**	**8.54E-11**	**1.37E-03**	**0.095**	**1**	**0.039**

Detailed examples of maximum likelihood analysis from samples NA12751 (7 copies), C0157 (9 copies) and NA12749 (5 copies) – see also Table 1. Although our analysis evaluates probabilities associated with copy numbers up to 16, for clarity we only show values up to 10 here. For sample NA12749, note that the absence of an indel5 ratio provides no information about likely copy numbers, and so all copy numbers are assigned an equal relative probability. The *DEFA1:DEFA3* ratio for NA12749 is most consistent with a copy number of 5, so that (on the basis of this measure alone) a copy number of 10 is equally well supported. Probabilities below about 10^-308^ are rounded to zero (see Additional file 1).

There was substantial variation in the confidence with which integer copy numbers were assigned, with MR ranging from just above unity (i.e., the assigned copy number was only marginally favoured over an alternative) to several million-fold. The median MR value was 20.1, and the interquartile range was 3.78-133.1; most samples, therefore, were assigned an integer copy number that was supported by a factor of at least 3 over alternatives. Low values of MR, corresponding to greater uncertainty in assignment to a particular integer, correlated as expected with (a) missing or uninformative data and (b) high copy number (see Additional file 1, Section 1d).

The analysis assumes that the same underlying copy number applies to all the sequence elements measured. To investigate whether any samples had evidence to the contrary, we highlighted samples as anomalous if they included one or more measure associated with a very low probability (P < 5 × 10^-4^) for the maximum-likelihood copy number. We found no evidence suggesting that any of the seven cases found in this way resulted from the existence of non-standard repeat units. Further discussion of this point can be found in the Additional file 1, Section 1d.

**Figure 3:**
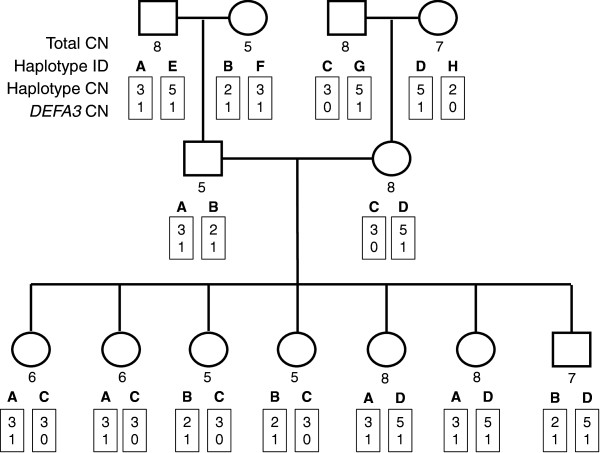
**Segregation of haplotypes in the three-generation CEPH family 1341.** In addition to the copy number, segregating haplotypes (boxed) are distinguished by the composition of other variants for which ratios were determined in this study. In this figure, only the *DEFA3* content of each haplotype is shown (as the figure at the bottom of the haplotype box), but other variants can also be used to distinguish and characterise the haplotype content. In this family, both parents possess a 3-copy haplotype (haplotypes A and C), but they are distinguished by the fact that only the haplotype (A) carried by the father contains a copy of *DEFA3*.

The distributions of diploid copy numbers for the two population samples examined (HapMap CEU [US] and ECACC [UK]) did not differ significantly (P > 0.2), and were therefore pooled to give an estimated distribution of copy numbers that should be generally applicable to white European populations (Table 3). At other multiallelic CNV loci, segregation has been powerful in validating the accuracy of copy number measurements, as well as in defining the constituent haplotypes of which the diploid CNV total is composed [29, 30]. We therefore examined the segregation of *DEFA1A3* copy number, of the inferred numbers of *DEFA1* or *DEFA3* alleles, and of indel alleles, in three-generation CEPH pedigrees (of which one example is shown in Figure 3). In 17 out of 23 families analysed in this way, all four parental haplotypes and their segregation could be inferred unambiguously from our observations alone, but in others haplotypes were resolved using the correct segregation pattern for the region as deduced from flanking SNP genotypes in CEPH families (http://www.cephb.fr/en/cephdb/browser.php), which were also used to confirm the grandparental origins of the parental haplotypes, and hence deduce the identities of the four grandparental haplotypes that were not transmitted to the parents. Determining frequencies of 179 haplotypes from segregation data allowed us to predict the expected frequencies for different diploid copy numbers under Hardy-Weinberg equilibrium. These predicted frequencies (Table 3) are not significantly different from those observed (P = 0.28).

**Table 3:** **Distribution of diploid copy numbers from 589 European samples typed in this work, and comparison with previous studies**

DEFA1A3 copy number	This study (N = 589)	Observed frequency	Predicted frequency (HWE)	Reconstructed from		
				Aldred (N = 111)	Linzmeier (N = 27)	Nuytten (N = 344)
≤3	1	0.002	0.004	0	0	0.006
4	28	0.048	0.045	0.027	0	0.037
5	69	0.117	0.129	0.144	0.037	0.192
6	117	0.199	0.177	0.261	0	0.372
7	121	0.205	0.215	0.243	0.111	0.257
8	129	0.219	0.205	0.198	0.111	0.087
9	64	0.109	0.113	0.108	0.111	0.043
10	37	0.063	0.085	0.009	0.259	0.006
11	13	0.022	0.017	0.009	0.074	0
12+	10	0.017	0.010	0	0.296	0

Distributions of diploid copy numbers in the 589 European samples typed in this work, and comparison with data taken or inferred from the previous studies of Aldred *et al.*[30], Linzmeier and Ganz [7], and Nuytten *et al.*[52]. The comparison is also made between the observed frequencies of copy number classes and those predicted from the haplotype frequencies determined in this study, assuming Hardy-Weinberg equilibrium (“Predicted frequency (HWE)”). The frequencies of copy number classes were not given explicitly by Nuytten *et al.*[52], but are reconstructed here from the data in their Figure seven (a). Jespersgaard *et al.*[47] do not give details of individual copy number counts, but instead give counts above or below a copy number of 6.

**Figure 4:**
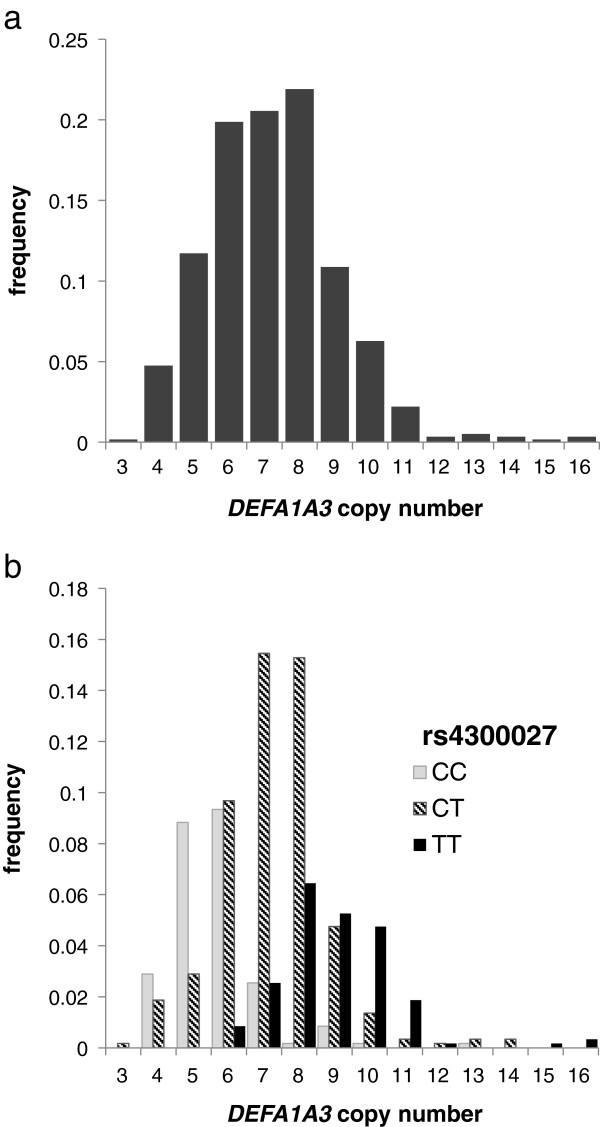
**Frequencies of measured integer** ***DEFA1A3*** **copy number states in Europeans. (a)** Histogram of copy number frequencies in 589 European DNA samples, **(b)** subdivided according to rs4300027 genotypes.

Comparison of haplotype copy numbers for CEU samples included in the HapMap project allowed us to investigate association between *DEFA1A3* copy number and local SNPs. The strongest association was with rs4300027, in a pattern corresponding to association of C at rs4300027 with 2- and 3-copy haplotypes, and T with 4- and 5-copy haplotypes. We therefore typed rs4300027 in the 589 samples for which we had determined copy number. A clear and strong association between rs4300027 and the copy number of alpha-defensin genes was confirmed, with the SNP genotype at rs4300027 approximately partitioning European samples into low (CC), medium (CT) and high (TT) copy number categories (Figure 4b: P = 1.3 × 10^-45^). This association is not absolute; some examples of exceptions to this correlation were observed, for example 4- and 5-copy individuals with rs4300027 genotype CT. These observations have been repeated and individually verified as genuine, and cannot therefore be attributed to inaccurate typing of copy number or of SNP genotype. Although the correlation illustrated in Figure 4b appears consistent, a (three-state) SNP genotype will always have limited ability to tag a multi-state CNV, and indeed the statistical power of rs4300027 genotype to predict *DEFA1A3* CNV status is indeed relatively modest (r^2^ = 0.35).

The definition of a SNP tagging *DEFA1A3* copy number allows us to perform indirect association tests by interrogating existing GWAS SNP data. If a clinical phenotype is strongly associated with *DEFA1A3* copy number, this should be indirectly reflected in an association with genotype at rs4300027, or the associated neighbouring SNPs rs4512398 (in near-complete LD with rs4300027 in European populations) and rs7825750 (r^2^ = 0.46 with rs4300027). Indeed, because of the strong but imperfect correlation with SNPs, a genuine underlying association with *DEFA1A3* copy number may be manifest in GWAS data as a P value (for example, in the range 10^-4^ to 10^-7^) too high to merit attention in a genome-wide context. Complete GWAS data, listing P values for all SNPs typed, were available from the WTCCC [35] and the CHAVI GWAS study of HIV control [36], and we obtained the assistance of relevant investigators in examining data from GWAS studies of atopic dermatitis [37], coeliac disease [38], Crohn’s disease [39, 40], type 1 diabetes [41], lung function in cystic fibrosis [42], multiple sclerosis [43], psoriasis [44, 45] and ulcerative colitis [40, 46]. These were interrogated for P values with rs4300027 or rs4512398 where genotyped, and rs7825750 in other studies. The results are collated in Additional file 1: Table S3, and reveal no strong indication of association with the *DEFA1A3* CNV as reflected indirectly in SNP data. It is noteworthy that for each of Crohn’s disease, psoriasis and type I diabetes there are two independent studies listed in Additional file 1: Table S3 that fail to show a significant association. Although the simplest explanation of these outcomes is that these phenotypes are not influenced by *DEFA1A3* copy number, even well-powered GWAS have limited power to positively exclude an association, especially at low effect sizes. Only coeliac disease (P = 0.013) demonstrated a P value below 0.05 (with rs4512398), but given that 18 different studies were examined, even that cannot be viewed as significant once a correction has been made for multiple testing (Additional file 1: Table S3). The relationship between CNV status and flanking SNPs might be different in different populations, and we therefore examined separately the largest single (UK) cohort in the study of Dubois *et al*. [38], consisting of 2586 cases of coeliac disease and 7532 controls; in this alternative analysis, the association with rs4512398 was not significant (P = 0.29).

We can therefore use these observations to suggest that a strong influence of *DEFA1A3* copy number on predisposition to any of these phenotypes in European populations is unlikely, despite the published evidence suggesting the influence of *DEFA1A3* copy number in Crohn’s disease [47] and of increased alpha-defensin production on HIV progression [48]. It also provides a simple (SNP-based) method for further investigation of other phenotypes in which *DEFA1A3* copy number may be implicated, such as the published association with sepsis [49], in a way that would not be complicated by the difficulties of direct copy number measurement. Nevertheless, although SNP genotyping can be used as an aid to prioritisation, because the association between rs4300027 and the CNV is imperfect, direct typing of the CNV remains the only definitive way to investigate potential associations.

## Discussion

In the absence of high-throughput methods that confer absolute assurance of gene copy number, detailed assessment of the accuracy of a new typing methodology is essential before it can be used in large sample sets. Having defined the copy number of some reference standard samples using definitive methods such as PFGE, these can be then used to calibrate and test further experiments. In addition, the evaluation of accuracy requires careful analysis of the internal consistency of data derived from the integration of different measurement assays. In principle, to achieve the best typing quality, large-scale association studies should ideally use pulsed-field gel analysis, but in practice few studies have the DNA resources, equipment and personnel to undertake the kind of exemplary work done at the complement C4 locus [50, 51]. In particular, wider replication of association findings generally depends on a reliable but high-throughput method to type DNA samples of the kind found in most population sampling studies.

Most *DEFA1A3* repeat alleles appear to harbour between 1 and 5 copies of a 19 kb copy-variable repeat, which allows different copy number alleles to be clearly distinguished after pulsed-field gel electrophoresis [30]. We were therefore able to use samples that had been definitively typed by this method [30] as the starting-point for calibrating our methods; subsequent analysis of segregation in three-generation pedigrees defined further reference samples that displayed unambiguous copy numbers on repeated testing using PRT and ratio methods (Figure 3). Larger-scale typing then produced data that were internally consistent between PRT and ratio measurements and conformed well to the predictions of Hardy-Weinberg equilibrium using haplotype frequencies determined in three-generation families. Reassurance of the correct calibration of our typing methods is particularly important given the apparent differences with the population copy-number distributions discovered by other approaches [7, 47, 52].

The copy-number frequencies found in this study are similar to those determined by Aldred *et al.*[30] who used a combination of MAPH and variant ratios, and although there are some differences (such as a higher frequency of copy numbers above 10 in the present work) the overall distribution is not significantly different (P = 0.073). By contrast, the differences between our data and the distribution given by Linzmeier and Ganz [7] based on real-time PCR measurements are highly significant (Table 3), especially in the representation of copy numbers above 8 (P = 1.95 × 10^-10^). Although it is possible that different population origins may influence the outcome, even the relatively small sample analysed by Linzmeier and Ganz seems incompatible with the values determined here, and may reflect limitations of real-time PCR typing for this locus. The study of Nuytten *et al.*[52] used real-time PCR calibrated against concatemeric constructs, but reports a copy number distribution that is also very significantly different from the one reported here (P = 1.1 × 10^-10^), with a much lower frequency of samples with copy numbers above 8. Nuytten *et al.* do not use reference genomic DNA standards, and despite their careful and ingenious method to calibrate real-time PCR measurements, it is possible that in this case their cloned constructs do not produce the same calibration as would be obtained from genomic DNA samples of the same copy number. The real-time PCR results from Danish samples given by Jespersgaard and colleagues [47] also have significantly more samples of low copy number (6 or fewer) among controls than we find in Europeans (P = 5.6 × 10^-3^), but not among their samples from Crohn’s disease patients (P = 0.074). Our preliminary analysis (data not shown) demonstrates a strong correlation with integer copy numbers published recently for HapMap Chinese and Japanese samples by Cheng *et al.*[53], although without further information on measurement variation or consistency for their real-time PCR assay it is not possible to judge the extent or causes of differences between our results.

In principle, read-depth analysis provides an alternative method to establish definitive diploid gene copy number for a sample, and the study of Sudmant *et al.*[54] first used genome-wide analyses of read depth to define copy number variation profiles for individual DNA samples. Although the available data suggest that their analysis of the *DEFA1A3* CNV is broadly comparable with ours (median copy number of 7.58 in Table S7 of Sudmant *et al.*, median value 7 in this study), no individual copy number values are given by Sudmant *et al.*, and their sample of 159 individuals comes from diverse global populations [54]. There were eight samples typed in our study which have also been sequenced as part of the Complete Genomics CNV Genome Baseline Set [55]. Our copy numbers for these samples have a strong correlation (r^2^ = 0.93) with the recorded sequence coverage (for further details see Additional file 1, Section 2). Microarray data for 108 HapMap samples from Campbell *et al.*[56] (their Supplementary Table S7) correlate reasonably well with our results (r^2^ = 0.49), even though the *DEFA1A3* CNV does not form discrete genotype classes in their analyses, and the absolute copy numbers are calibrated by comparison of microarray signals against single-copy regions rather than specifically against known *DEFA1A3* copy numbers. Presumably for this reason, Campbell *et al.* report copy number ranges for *DEFA1A3* higher than measured in this study (mean 9.5 and median 9.4, compared with 7.5 and 8 respectively in this study). These analyses are described in Additional file 1, Section 2, and illustrated by a scatterplot in Additional file 1: Figure S4. Although the *DEFA1A3* CNV was not called individually in the 42 million-element array-CGH study of Conrad *et al*. [57], their publicly available data can be compared with our own results for 17 samples, in which a good correlation (r^2^ = 0.74) is found (see Additional file 1, Section 2, and Additional file 1: Figure S5). The CNV at *DEFA1A3* does not seem to have been defined and analysed in other recent studies on genome-wide identification of CNVs through read-depth analysis [58, 59].

By comparison with flanking SNP genotypes in HapMap samples we were able to define a strong association between *DEFA1A3* copy number and rs4300027. To a first approximation this single SNP partitions our samples into classes with low (up to 6 copies), medium (6 to 8 copies) and high (8 copies or more) copy number, although initial further work suggests that this is not a simple cladistic split into high- and low-copy lineages (data not shown). In addition to its practical power in exploring possible associations of *DEFA1A3* copy number with disease phenotypes, the strength and consistency of this association provides additional reassurance that our copy number typing is not subject to wide variation in accuracy. It is important to note that the samples analysed here are of European origin, and so rs4300027 can be used with confidence as a surrogate for *DEFA1A3* copy number only in European cohorts. Most published GWAS data sets do indeed analyse European subjects, but our initial exploration of the HapMap samples suggests that the strong association of rs4300027 with copy number is not reproduced in Asian or African populations.

## Conclusions

We have developed a PCR-based methodology for copy number measurement of the human alpha-defensin *DEFA1A3* gene cluster. Our data show good internal evidence of accuracy and consistency, and we have discovered that *DEFA1A3* copy number is strongly associated with SNP rs4300027 in European samples. This has in turn led to the application to GWAS investigations of rs4300027 genotype as a good proxy for approximate copy number range in Europeans.

## Methods

### DNA samples and standards

180 CEPH samples from the International HapMap phase I and II (http://ccr.coriell.org) and 480 random UK samples from the European Collection of Cell Cultures (ECACC) Human Random Control (HRC) panels 1 to 5 (http://www.hpacultures.org.uk) were used to develop the copy number measurement assays. The CEPH (CEU) samples used consist of 56 family trios, 5 duos and 2 singletons. For the data presented in the Results, only the 120 unrelated HapMap CEU samples were considered, so that we attempted to type 600 unrelated European samples, of which 589 produced satisfactory results. A further 110 individual CEPH samples were used to infer segregation of the CEPH trios from HapMap samples and another 99 individual CEPH samples from 3-generation pedigrees not included in the HapMap project were also used for segregation. The 23 CEPH families for which further samples were available and thus allowed segregation were; 12, 66, 104, 884, 1331, 1332, 1333, 1334, 1340, 1341, 1344, 1345, 1346, 1350, 1362, 1375, 1408, 1416, 1420, 1421, 1424, 1454, 13292. All DNA provided was extracted from lymphoblastoid cell lines.

In initial development our typing methods were calibrated using the reference samples of known *DEFA1A3* copy number defined by Aldred *et al.*[30] after pulsed-field gel electrophoresis and Southern blotting. These samples were used to define a second set of reference samples, this time from publicly-available sources. After initial calibration against the original pulsed-field gel-typed reference samples, the copy numbers of these new reference samples were confirmed by consistency of numerous repeated measurements using different methods, and by analysis of segregation within three-generation families (see Results below). The data reported in this paper were obtained by calibration against these new reference samples. The new reference samples were four samples available from the ECACC HRC-1 collection, C0007 (7 copies), C0075 (6 copies), C0150 (8 copies) and C0877 (9 copies), with three offspring from CEPH pedigrees (DNA available from Coriell), NA07062 (=1340-3, 5 copies), NA11998 (=1420-4, 6 copies) and NA07008 (=1340-5, 7 copies).

### PCR and PRT methods

All PCR used 10 ng of input DNA, and a standard buffer at a final concentration of 50 mM Tris–HCl (pH8.8), 12.5 mM ammonium sulphate, 7.5 mM 2-mercaptoethanol, 125 μg/ml BSA, 1.4 mM MgCl_2_, and 200 μM each dNTP. PCR products were denatured in 10 μl formamide containing ROX-500 markers (Life Technologies) before denaturation (96°C, 3 minutes) and capillary electrophoresis. Although other combinations are possible, our work combined 1 μl each of FAM- and NED-labelled MLT1A0 PRT products with 1 μl of indel5, followed by electroinjection at 1 kV for 30 seconds into an ABI 3130xl Genetic Analyzer. Similarly, 4 μl of *Msp*I-digested *DEFA4* PRT PCR product and 4 μl *Hae*III-digested DefHae3 PCR product were added to 10 μl formamide/ROX mixture, with injection at 2 kV for 45 seconds. GeneMapper software (Applied Biosystems) was used to extract the peak areas of the separated PCR products.

MLT1A0 PRT was performed using two independent PRT assays, one with a FAM labelled forward primer and the other with a NED labelled forward primer, that are then averaged into a single unrounded copy number value. Each PCR was performed with 1 μM each of primers (FAM/NED)-CCCAGAGAGCTCCTTC and GTGACTTATAAACAACAAAAA, using 24 cycles of 95°C for 30 seconds, 48°C for 30 seconds and 72°C for 30 seconds, followed by a 10-minute hold at 72°C. The primers amplified from an MLT1A0 dispersed repeat present in full repeats (only, see Figure 1) at *DEFA1A3* and a similar repeat at the reference locus on chromosome 1. The MLT1A0 PRT gives products of 170 bp for the reference locus on chromosome 1 and 167 bp for the full repeat region of *DEFA1A3*.

*DEFA4* PRT used 1 μM primers TGCTCCTGCTCTCCCTCCT and (HEX)- TTGGAATCAAGTCTTTGGAGAAA, amplifying for 26 cycles of 95°C for 30 seconds, 56.5°C for 30 seconds and 70°C for 30 seconds, followed by a 70°C hold for 10 minutes. This PCR exploits sequence similarities between the closely related genes *DEFA1A3* and *DEFA4*, such that the primers were specifically designed to match sequences in both genes, giving products of 404 bp for the reference locus and 406 bp for *DEFA1A3*. These products cannot be completely separated by electrophoresis, and therefore an overnight restriction digestion at 37°C by *Msp*I was performed which gives labelled products of 275 bp for the *DEFA4* reference locus and 317 bp for *DEFA1A3*. Although we have observed a single instance of a haplotype carrying a deletion of *DEFA4*, no further examples of this variant have been observed (see Additional file 1, section 1(d)).

### Ratio methods

The ratio between the *DEFA1* and *DEFA3* gene variants was measured using an assay (“DefHae3”) exploiting the *Hae*III restriction site difference between them. PCR used 1 μM of primers TGTCCCAGGCCCAAGGAAAA and FAM- TCCCTGTAGCTCTCAAAGCA, using 25 cycles of 95°C for 1 minute, 58°C for 1 minute and 70°C for 1 minute, followed by a 70°C hold for 10 minutes. The underlined base in the forward primer is a deliberate mismatch with the genomic sequence to create an artificial site for *Hae*III. Because a completely undigested product arising from incomplete activity of the restriction enzyme cannot otherwise be distinguished from the (*DEFA3*) variant lacking an internal *Hae*III site, it was necessary to introduce this artificial site into all products to act as a check of complete digestion by *Hae*III. *DEFA1* (*Hae*III+) products yield a labelled product of 144 bp, and *DEFA3* (*Hae*III-) products 161 bp. PCR product (5 μl) was digested with 1.5U *Hae*III in a total volume of 15 μl at 37°C for 12-16 hours. The full-length PCR product, indicating incomplete digestion, would be 170 bp. The DefHae3 ratio recorded is the ratio of 144 bp to 161 bp products, i.e., the *DEFA1:DEFA3* ratio.

A deletion variant present in many repeats formed the basis of the “indel5” ratio assay. Indel5 PCR used 1 μM of primers HEX-CTGTCCAGGAAGAGGGAGAG and CAGCTGGAGGGTCTCTGTTC, and 23 cycles of 95°C for 30 seconds, 57°C for 30 seconds and 70°C for 30 seconds, followed by a 70°C hold for 10 minutes to generate amplicons of 124/129 bp. The indel5 ratio recorded is the ratio of deleted (124 bp) to undeleted (129 bp) products.

A 7 bp duplication variant present in many repeat units provided the basis of a third (“7bpdup”) ratio measurement. This assay used primers (HEX)- AGCAAAAATCAAACAACCTGA and GCTATGCCTCCAATCTGACC; after an initial denaturation of 95°C for 1 minute, products were amplified for 24 cycles of 95°C for 30 seconds, 54°C for 30 seconds and 70°C for 30 seconds, followed by a final hold at 70°C for 40 minutes. The 7bpdup ratio recorded is the ratio of unduplicated (275 bp) to duplicated (282 bp) products.

### Genotyping of SNP rs4300027

Genotyping of rs4300027 was performed by PCR-RFLP. A single PCR reaction was performed with 1 μM each of primers AGATACCATGCTTGGAGGAA and GGGTCTTGAATTCAAATGTCAG. PCR cycle conditions were 36 cycles of 95°C for 30 s, 58.6°C for 30 s, 70°C for 30 s to generate an amplicon of 1043 bp in length. In the *C allele this is cleaved by *Hin*fI to produce 6 fragments of 439 bp, 174 bp, 154 bp, 116 bp and 105 bp, as well as a small fragment of 55 bp. The second cleavage fails to occur in the presence of the *T allele and so a product of 613 bp is observed, as well as the other small (154 bp, 116 bp, 105 bp and 55 bp) fragments. The distinction between the longer allelic digestion fragments of 439 bp and 613 bp is clearly visible on a 2% (w/v) agarose gel.

### Data analysis

PRT ratios were used to estimate gene copy number values, calibrating against reference samples of known copy number as described [24]. These PRT copy number values were combined with ratio values for the same sample (from the indel5, *DEFA1*: *DEFA3* and 7bpdup ratio measurements) to evaluate the most likely individual integer gene copy number. Briefly, for each PRT or ratio measurement, Gaussian models of measurement error (based on empirical observations) were used to estimate the probability of producing the actual measurement, given a particular value for the true gene copy number between 2 and 16. Once these probabilities had been determined for each measurement at each copy number, they were combined by multiplication to identify the integer copy number that maximises the joint probability of all the data, the “maximum likelihood copy number (MLCN)”. Further details can be found in Additional file 1.

For inclusion in the analysis, a sample needed to have at least two non-zero data points, of which at least one was a PRT. Out of the 600 DNA samples initially tested, 589 (98.2%) met these criteria.

### Availability of supporting data

The data sets supporting the results of this article are included within the article (and its additional files).

## Electronic supplementary material

Additional file 1: **Further details on data analysis, including Supplementary Figures and Tables.** More detailed explanation and details are given about the methods used in analysing the data (Section 1), and on comparison with read-depth data from Complete Genomics (Section 2). (PDF 577 KB)

Additional file 2: **Measured values for 589 European samples.** This file shows the measurements obtained for the 589 samples examined in this study. The PRT values shown (MLT1A0 PRT and DEFA4 PRT) are unrounded copy number estimates after calibration against reference standards. The ratios (indel5, DefHae3, 7bpdup) are shown as the raw uncalibrated ratios derived directly from the peak measurements. In this datafile (and Additional file 3) “0” is used as a catch-all indicator of the absence of information – either because no result was obtained, or because only one of the two peaks was present for the ratio measurements. In no case was a true value of 0 ever actually measured for either PRT. (CSV 36 KB)

Additional file 3: **Maximum-likelihood analysis of data from Additional file** 2 **.** This file shows the maximum-likelihood analysis results for the same 589 samples based on the input data shown in Additional file 2. For each sample in turn, the input data are listed in column B, and the Maximum-Likelihood Copy Number (MLCN) and minimum ratio in columns C and D. The remaining columns show the individual relative probabilities for each putative copy number based on each of the measurements, with the “Combined” line at the top of each group giving the compound probabilities. As in Additional file 2, a value of “0” can indicate either missing data or an uninformative outcome, rather than a measured copy number of 0. Probability values below about 10^-308^ are rounded to zero. (CSV 549 KB)
