# Supplementary material for: Accurate measurement of gene copy number for human alpha-defensin DEFA1A3
Source: BMC Genomics. 2013 Oct 20;14(1):719. doi: 10.1186/1471-2164-14-719 (PMC4046698; doi:10.1186/1471-2164-14-719)
Supplement: Supplementary file 1 — Additional file 1: Further details on data analysis, including Supplementary Figures and Tables. More detailed explanation and details are given about the methods used in analysing the data (Section 1), and on comparison with read-depth data from Complete Genomics (Section 2). (PDF 577 KB) [file 12864_2013_5441_MOESM1_ESM.pdf]

Additional File 1: Supplementary Information

Section 1: Details of measurement and analysis methods

(a) Maximum-likelihood methods

The maximum-likelihood approach to copy number determination we adopted was similar in its general structure to that used by Aldhous *et al*. for beta-defensins [1], and our rationale for adopting such an approach was two-fold. Firstly, we needed a way of combining information from different independent measures of *DEFA1A3* copy number. This was particularly important in allowing us to leverage the power of the allele ratio measurements, which we have found to have high empirical accuracy, presumably because allele ratios do not compare representation of the target sequence with another (reference) locus in the genome, but instead compare variants of the target sequence with each other. Nevertheless, allele ratio methods alone are not sufficient – a measured allele ratio of 2:1 is compatible with true copy numbers of 3, 6, 9 or any multiple of 3 – and PRTs (or some other method of fixing the approximate copy number relative to the genome as a whole) are necessary to arrive at a reliable copy number determination. If all the data were PRT measurements, we could simply combine the measurements as an average (or weighted average if differences in accuracy need to be taken into account); by contrast, allele ratios and PRTs are very different types of data, and likelihood evaluations allow us to combine them on an even basis.

The second rationale was that a likelihood approach would have a built-in method for evaluating the confidence that could be placed in an integer copy-number call. Although the distributions of error around each measurement are not known *a priori*, we have enough empirical data from samples of confidently-assigned copy number to model the expected distribution of measurements, and these can in turn be used to derive the probability of the observed measurement conditional on a particular true integer copy number. The distributions observed (see Supplementary Figure 1 for the PRT measurements) approximate quite well to a Gaussian, and despite a small excess of values at the extremes of some distributions (Supplementary Figures 1 and 2), they fit well enough in the central range $-2 < z < 2$ in which most observations are found that we adopted a Gaussian model as mathematically convenient.

(b) Evaluating likelihoods

For the PRT measurements, the probability of measuring the value observed assuming a true underlying copy number was determined assuming that PRT measurements of samples of true copy number N would have a Gaussian distribution with mean N, and a standard deviation estimated from repeated analysis

of samples of known copy number. Using a relatively small number of reference standards allowed us to estimate standard deviations for PRT measurements that gave useful analyses, but appeared to underestimate the standard deviations for all copy-number classes relative to the data observed in a larger data set. Further analysis of PRT measurements showed that higher standard deviations were observed in analysis of many different samples of the same copy number relative to repeated analysis of the same samples. We therefore increased the estimated standard deviations to take account of this phenomenon, resulting in analyses that were more internally consistent. This procedure was also conservative, in the sense that it would act to underestimate the overall confidence in our integer copy number calls. We have never observed a sample with a copy number of 2, and there are few each of copy numbers above 11 in our dataset. For these copy numbers, standard deviations were estimated by extrapolation from neighbouring values. The standard deviations used in our analyses were:

CN	MLT1A0 PRT	DEFA4 PRT
2	0.2	0.2
3	0.3	0.3
4	0.43	0.54
5	0.53	0.65
6	0.63	0.76
7	0.73	0.87
8	0.83	0.98
9	0.93	1.09
10	1.03	1.2
11	1.3	1.3
12	1.4	1.4
13	1.6	1.6
14	1.8	1.8
15	2	2
16	2.2	2.2

For the ratio methods, because the same copy number could be informatively represented by many compatible integer splits (a copy number of 7 could be split 0:7, 1:6, 2:5, 3:4, 4:3, 5:2, 6:1 and 7:0) we analysed all possible splits for each copy number, analysing for each copy number the split that best matched the observed ratio. This best-fitting observed ratio was then expressed as a normalised ratio, the ratio of the observed value relative to the expected; for example, a measured ratio of 1.8 and a MLCN of 6 (for which the best-fit integer ratio is 4:2, or a ratio of 2.0) would generate a NR of 0.9. Normalised allele ratios appeared to have empirical distributions that approximate well to Gaussian distributions (see below). Our data allowed us to observe that ratio measurements for indel5 and DefHae3 had normalised values that were consistently different from the expected mean value of

1.0 (see below), and the data allowed us to estimate standard deviations for the normalised values.

(c) Observed measurement distributions

Supplementary Figure 1 shows the observed distributions of normalised ratio (NR) values, and Supplementary Figure 2 shows the quantile-quantile plots against a Gaussian distribution. For PRTs, the normalised value is the observed PRT measurement expressed relative to the maximum-likelihood copy number (MLCN, see below) for that sample. Thus a PRT measurement of 6.6 in a sample to which an MLCN of 6 was assigned would generate a normalised ratio of 1.1. For the allele ratios, the NR is the measured ratio expressed relative to the best-fitting integer split of the MLCN (see above).

Previous experience [2] had shown that the DefHae3 (*DEFA1:DEFA3*) ratio test had a tendency to overestimate the representation of *DEFA3* by about 10%. On average, samples with equal numbers of *DEFA1* and *DEFA3* genes would record a *DEFA1*:*DEFA3* ratio of about 0.92, which we assume is due to the generation of heteroduplex DNA that would fail to digest with *Hae*III (see Supplementary Figure 2d, below). In our analysis, we corrected for this bias by modelling a distribution of NR values with a mean of 0.918 rather than 1. Similarly, presumably because the shorter PCR product has a small amplification advantage relative to the longer one, the ratio of the deleted to undeleted forms of the indel5 variant has a mean of about 1.05, which was used as the mean of the NR distribution.

(d) Minimum ratios as confidence measures

We used the likelihood estimates to determine a measure of the confidence with which a MLCN was assigned – in some cases there would be multiple measurements strongly supporting the same MLCN, leading to high confidence, whereas in other cases some of the measures might be missing, uninformative or contradictory, leading to lower confidence in the best-supported single integer value for the MLCN. We defined the “minimum ratio” (MR) as the factor by which the likelihood associated with the MLCN exceeded the next best supported integer copy number. In our data, these MR values had a median value of 20.1 and an upper quartile value of 133.1, suggesting that most samples had a strongly supported MLCN, but there were also many samples with relatively low MRs (the lowest quartile value is 3.78). There was a strong association between low MR and missing or uninformative data – the 528 samples with four or five informative values out of the five possible measures (two PRTs and three ratios) had a median MR of 25.48, while the 61 samples with three or fewer informative measurements had a median MR of 4.65. A technically accurate ratio measurement can be uninformative if only

one of the two variants is present – this is particularly frequent for the DefHae3 ratio, for which about 10% of the population lack *DEFA3* [2]. Low MR was also strongly correlated with high copy number, presumably because of the increased difficulty in distinguishing neighbouring integer values; of the 336 samples with MLCN values between 3 and 7, the median MR was 93.51, whereas the 253 samples with MLCN of 8 or more had a median MR of 5.12.

In most cases the data supported an interpretation that all measurements were consistent with a single underlying copy number value, but we wanted to ensure that we were aware of any evidence that any of the tests was measuring a value genuinely inconsistent with the consensus. This might happen either because of variant repeats with altered representation of the measured sequences, unexpected SNP variation compromising the efficiency of PCR from particular targets, or because of copy number variation of reference loci in PRT assays. We therefore screened our analyses to identify any samples for which the relative P value for the MLCN (derived from integrating all measurements) was below 0.0005 for any individual test. Because we obtained five measures of copy number for each of more than 500 samples, we considered that a threshold of 1 in 2000 would allow us to identify any strong anomalies without creating too great a burden of false positives.

Inspection of the 7 samples (1.2%) highlighted as anomalous showed no recurrent pattern of discrepancy that explains most of the anomalies. All these samples did show unusually divergent values between the two PRT measurements, which might in principle indicate reference locus variation. We therefore retyped the PRTs for these samples, which yielded values on retyping that were more consistent with each other and with one of the previous PRT measurements. We would therefore suggest that our initial measurements of these samples represent extremes of random measurement variation, and are not evidence of uncommon genotypes that lead to systematic error in copy number evaluation.

One sample (NA12045) included in our sample set was known from other work [3] to carry a heterozygous deletion of *DEFA4*, such that the DEFA4 PRT would be expected to return a copy number estimate (based on the assumption that two copies of *DEFA4* were present) of about twice the true value. This deletion was tracked using a PCR assay in CEPH pedigree 1346, and is found in the mother (CEPH 134602 = NA10852), her father (CEPH 134613 = NA12045) and four offspring. Although NA12045 is not highlighted as anomalous in this work by the analysis program, the DEFA4 PRT does return a value (18.95) much higher than the MLT1A0 PRT value (13.58); these measurements constitute the extreme outlier point at the top right of Figure 2. If this deletion of *DEFA4* were prevalent, it would compromise the general applicability of our combined PRT scheme for *DEFA1A3* copy number measurement. We therefore used the PCR-based assay to type the *DEFA4* deletion status of all samples flagged as anomalous in the analysis (see above) and samples with DEFA4 PRT copy number values more than 1.4 times the MLT1A0 value. We found no further examples of the *DEFA4* deletion, and conclude

that it is present at low frequency; even with incomplete ascertainment, since one heterozygous example was found in 589 unrelated individuals, the allele frequency is likely to be of the order of 0.001.

Our maximum likelihood analysis does not take into account any prior probabilities derived from known distributions of copy number within the population; this assumption of "flat priors" is necessary in this initial analysis, to avoid circularity of argument, but the analysis could be extended in future work by applying known frequencies of particular diploid copy number states as prior probabilities. This would have the effect of increasing the confidence of assignment (as measured by the Minimum Ratio) for the majority of common copy number states.

We applied the analysis procedure in the context of a custom program written in c++ (source code available on request). In this context, the smallest positive number that can be represented as a double-precision variable is about 10^{-308}, and probability values smaller than this are rounded to zero.

Section 2: Comparison with other *DEFA1A3* CNV measurements

We were able to compare our measurements with eight samples that were both part of our sample set and included in the CNV Genome Baseline Set of Complete Genomics [4]. As the read-depth most likely to correspond to *DEFA1A3* copy number, we selected sequence bins (chr8: 6822000-6828000, 6840000-6848000 and 6860000-6866000) that included the gene sequences and surrounding sequences shared at high levels of sequence identity between the repeats on the human genome assembly (Build 36). The mean values across these intervals for median-scaled, GC-corrected read depth representation are shown relative to the corresponding MLCN values in Supplementary Table 1 below, and in Supplementary Figure 3.

The comparison with the microarray data of Campbell *et al*. used the values from their Table S7 for the “non-discrete” CNV defined by 52 probes in the interval chr8:6815835-6866374. There were 108 samples typed for that CNV by both Campbell *et al*. and in this study, with an r^2 value of 0.49 between the different measures. A scatterplot of the results is shown as Supplementary Figure 4, below. Using publicly available intensity data for 17 samples common to both studies (http://www.sanger.ac.uk/research/areas/humangenetics/cnv/highres_discovery.html) from the 42 million-element array-CGH data of Conrad *et al.* (2010), the median $\log_2$ ratio values across the three repeat intervals at chr8:6,822,000-6,828,000, chr8:6,840,000-6,848,000 and chr8:6,860,000-6,866,000 were compared with the MLCN from this study. A scatterplot of these comparisons is shown in Supplementary Figure 5 below (r^2 = 0.74). Although we have not determined its copy number directly in this study, these comparisons are consistent with the reference sample NA10851 having a copy number of 7.

Section 3: P values from GWAS studies

After consulting with the relevant investigators, we interrogated 18 GWAS studies of infectious/inflammatory disorders for P values at SNP loci associated with *DEFA1A3* copy number, and Supplementary Table 3 below summarises those findings. In addition to the GWAS studies of inflammatory conditions specifically interrogated in that approach, further P values for rs4300027, rs4512398 and rs7825750 can be obtained by searching GWAS Central (http://www.gwascentral.org/index). In total, 152 entries were found in GWAS Central for P values involving these SNPs. Among these 152 entries, there were 6 examples of P values below 0.05:

SNP	P value	phenotype	GWAS Central ID
rs4512398	0.007237	Rheumatoid arthritis	HGVST185
rs4512398	0.008682	Parkinson's disease	HGVST6
rs7825750	0.0174983	Systolic blood pressure	HGVST307
rs4512398	0.02662	Parkinson's disease	HGVST6
rs4512398	0.03606	Rheumatoid arthritis	HGVST185
rs4300027	0.03989	Amyotrophic lateral sclerosis	HGVST65

The first and fifth entries, and the second and fourth, are variant analyses of the same SNP in the same data set, and are therefore not independent. These include no examples of P values that are of clear significance given the number of values considered overall. Taken together the P values from non-duplicate SNP analyses conform well to the expectation for 87 values randomly distributed between 0 and 1 (see Supplementary Figure 6, below).

Supplementary Tables

Supplementary Table 1

DEFA1A3 MLCN (this study) and median-scaled, GC-corrected read depth (Complete Genomics) for eight samples common to both studies.

sample	MLCN	read depth
NA06985	7	2.26
NA06994	6	1.94
NA07357	10	3.33
NA12004	7	2.27
NA12889	10	2.87
NA12890	5	1.69
NA12891	5	1.73
NA12892	7	2.51

Supplementary Table 2

New reference standards used in this study. “CEPH family ID” is given for those samples which have been validated not only via repeated concordant measurements against previous reference samples (of copy number deduced from restriction fragment lengths established by Southern blotting of pulsed-field gels), but also from segregation of haplotypes established via segregation in three-generation CEPH pedigrees. These particular copy number values can therefore be regarded as very strongly established.

**Sample**	***DEFA1A3* CN**	**Source**	**CEPH family ID**
C0007	7	ECACC HRC1	
C0075	6	ECACC HRC1	
C0150	8	ECACC HRC1	
C0877	9	ECACC HRC1	
NA07062	5	CEPH	1340-3
NA11998	6	CEPH	1420-4
NA07008	7	CEPH	1340-5

Supplementary Table 3

Summary of P values for *DEFA1A3* CNV-associated SNPs in 18 independent GWAS studies of inflammatory or infectious disorders. In the single instance of a nominally significant point P value ($P_{obs} < 0.05$), the adjusted P value (P_{corr}) is shown using a Bonferroni correction for the 18 different studies analysed: $P_{corr} = 1 - [(1-P_{obs})^N]$, where N is the number of tests, which approximates to NP_{obs} when N is large and P_{obs} small.

Phenotype	SNP	P_{obs}	P_{corr}	Reference
**WTCCC**				
Bipolar disorder	rs7825750	0.529		[5]
Coronary artery disease	rs7825750	0.555		[5]
Crohn’s disease	rs7825750	0.168		[5]
Hypertension	rs7825750	0.660		[5]
Rheumatoid arthritis	rs7825750	0.365		[5]
Type 1 diabetes	rs7825750	0.201		[5]
Type 2 diabetes	rs7825750	0.191		[5]
Coeliac Disease	rs4512398	0.013	0.21	[6]
type 1 diabetes	rs4512398	>0.05		[7]
	rs4300027	>0.05		[7]
Ulcerative colitis	rs4512398	0.8		[8]
	rs4300027	0.71		[8]
Crohn’s Disease	rs4512398	0.525		[9]
IBD	rs4512398	>0.05		[10]
	rs4300027	>0.05		[10]
Psoriasis	rs4512398	0.2		[11]
Psoriasis	rs4300027	0.49		[12]
	rs4512398	0.926		[12]
Atopic Dermatitis	rs7825750	>0.05		[13]
Multiple Sclerosis	rs7825750	0.5904		[14]
HIV progression	rs4300027	0.946		[15]
	rs4512398	0.783		[15]
	rs7825750	0.79		[15]
CF severity	rs4300027	0.10		[16]
	rs4512398	0.12		[16]

Supplementary Figures

Supplementary Figure 1

Normalised Ratio (=measurement/MLCN) distributions for (a) MLT1A0 and (b) DEFA4 PRTs.

(a)

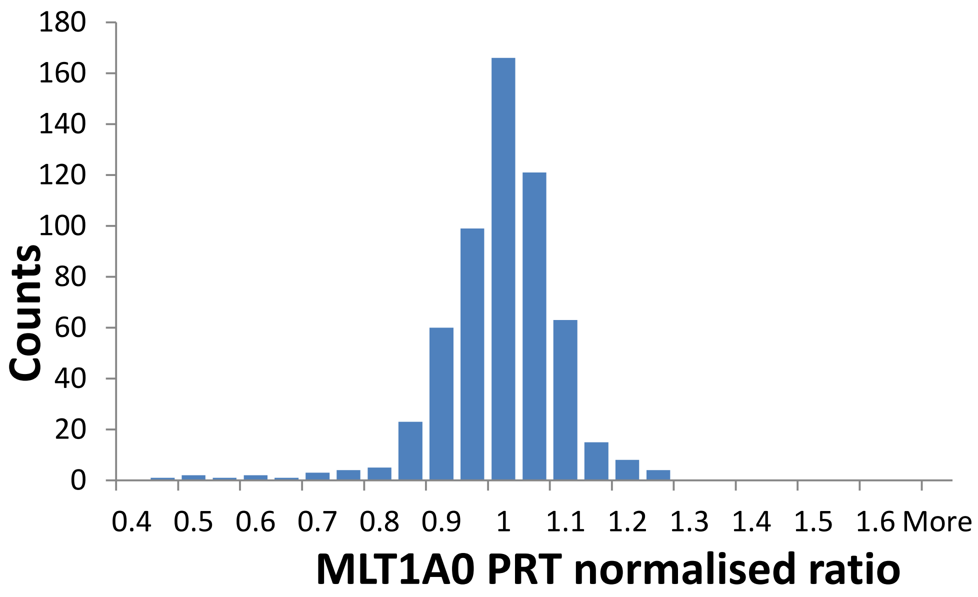

(b)

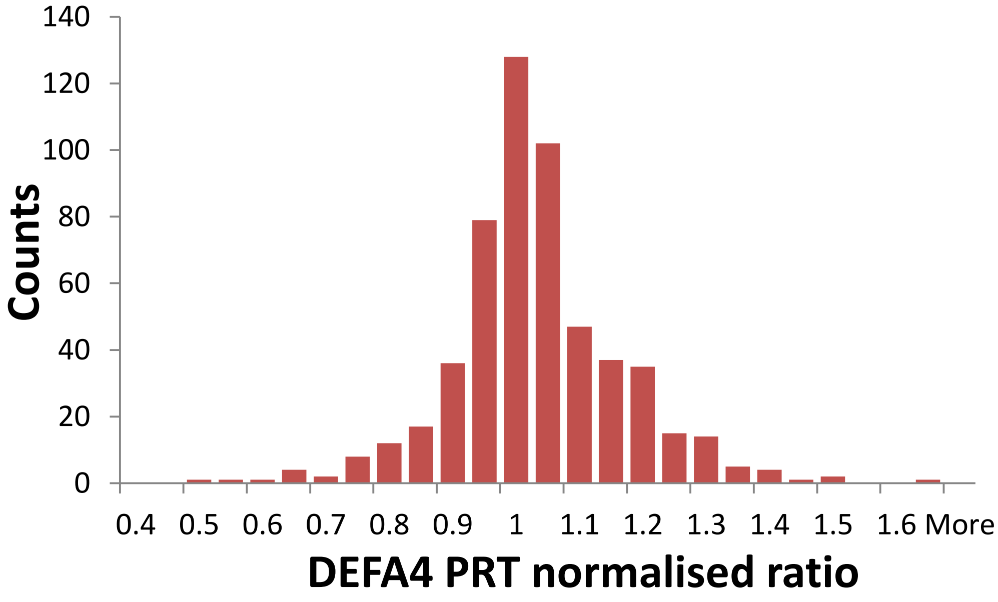

Supplementary Figure 2

QQ plots of NR relative to a Gaussian distribution for all 5 measures: (a) MLT1A0 PRT, (b) DEFA4 PRT, (c) indel5 ratios, (d) DefHae3 (*DEFA1*:*DEFA3*) ratios and (e) 7bp duplication ratios. For a given MLCN, in (c)-(e) there will more than one possible split of variants consistent with the integer total, and the ratio that most closely matches the observed value has been used.

(a)

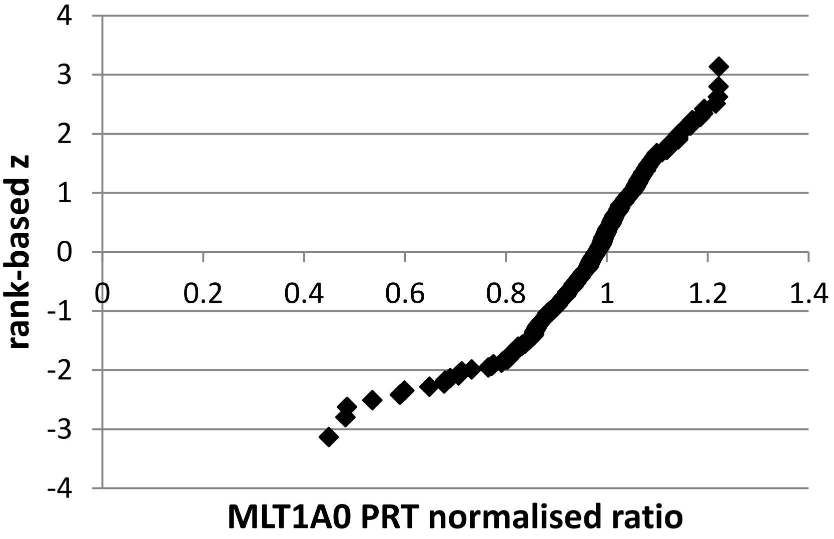

(b)

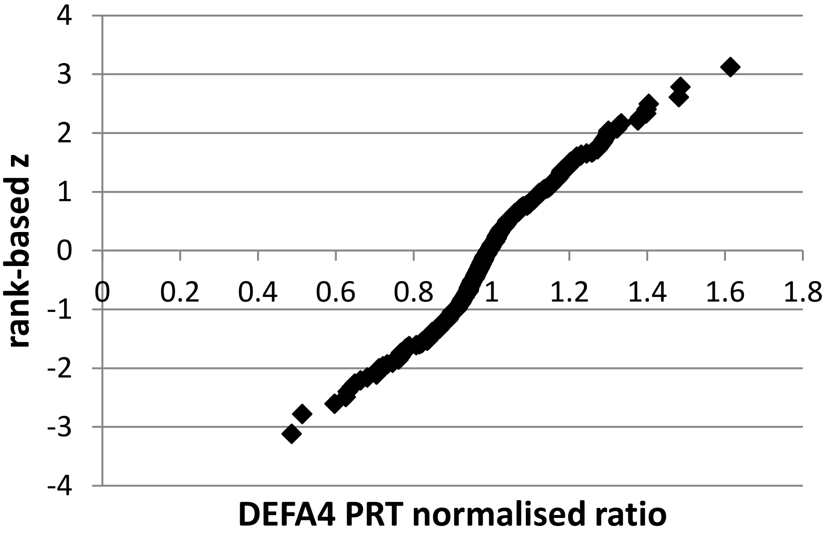

(c)

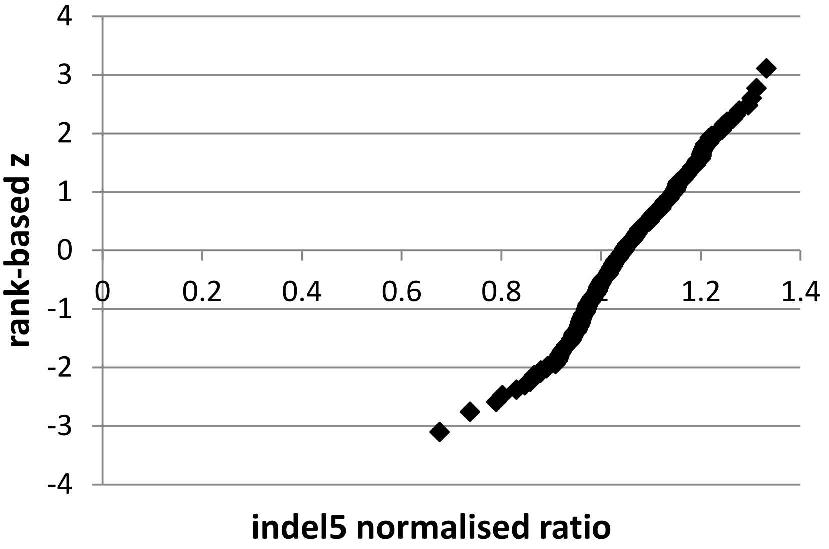
4
3
2
1
0
-1
-2
-3
-4
0
0.2
0.4
0.6
0.8
1.2
1.4
rank-based z
indel5 normalised ratio

(d)

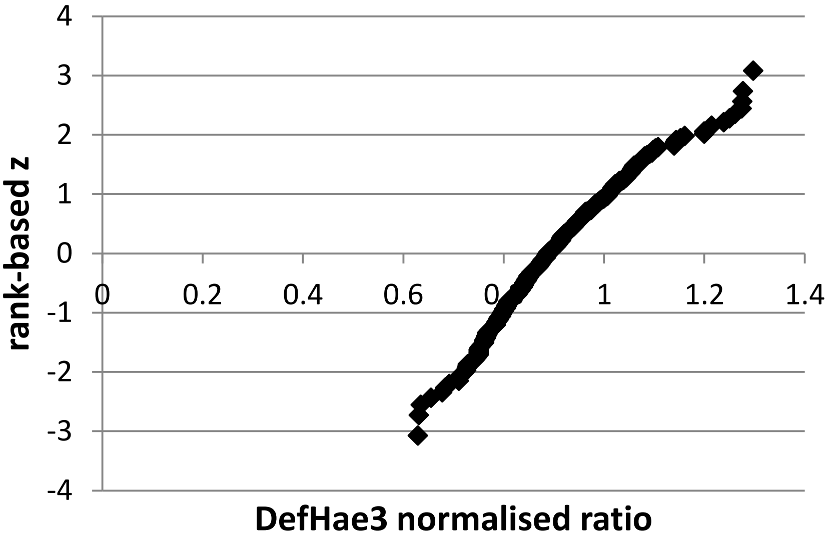
4
3
2
1
0
-1
-2
-3
-4
0
0.2
0.4
0.6
1
1.2
1.4
rank-based z
DefHae3 normalised ratio

(e)

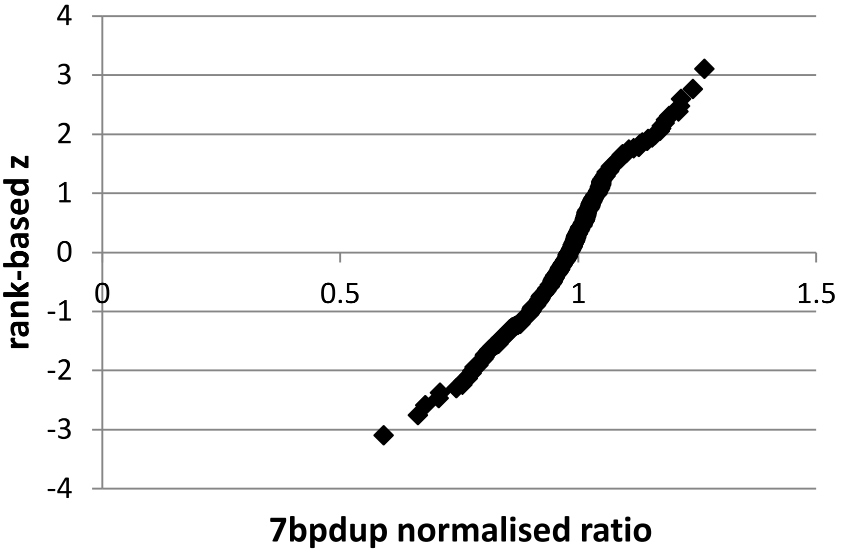

Supplementary Figure 3

DEFA1A3 MLCN (this study) plotted against median-scaled, GC-corrected read depth (Complete Genomics) for eight samples common to both studies (see also Supplementary Table 1).

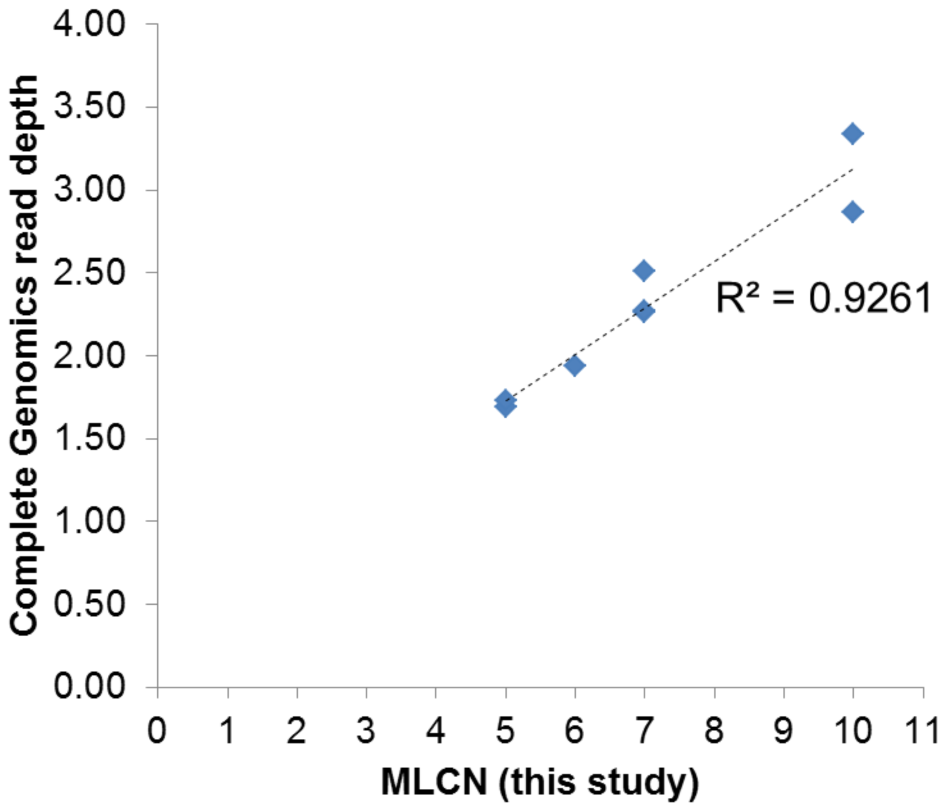

Supplementary Figure 4

Scatterplot comparing the MLCN values in this study with the microarray data of Campbell *et al*. (their Table S7, chr8:6815835-6866374) for 108 HapMap samples typed in both studies ($r^2 \approx 0.49$).

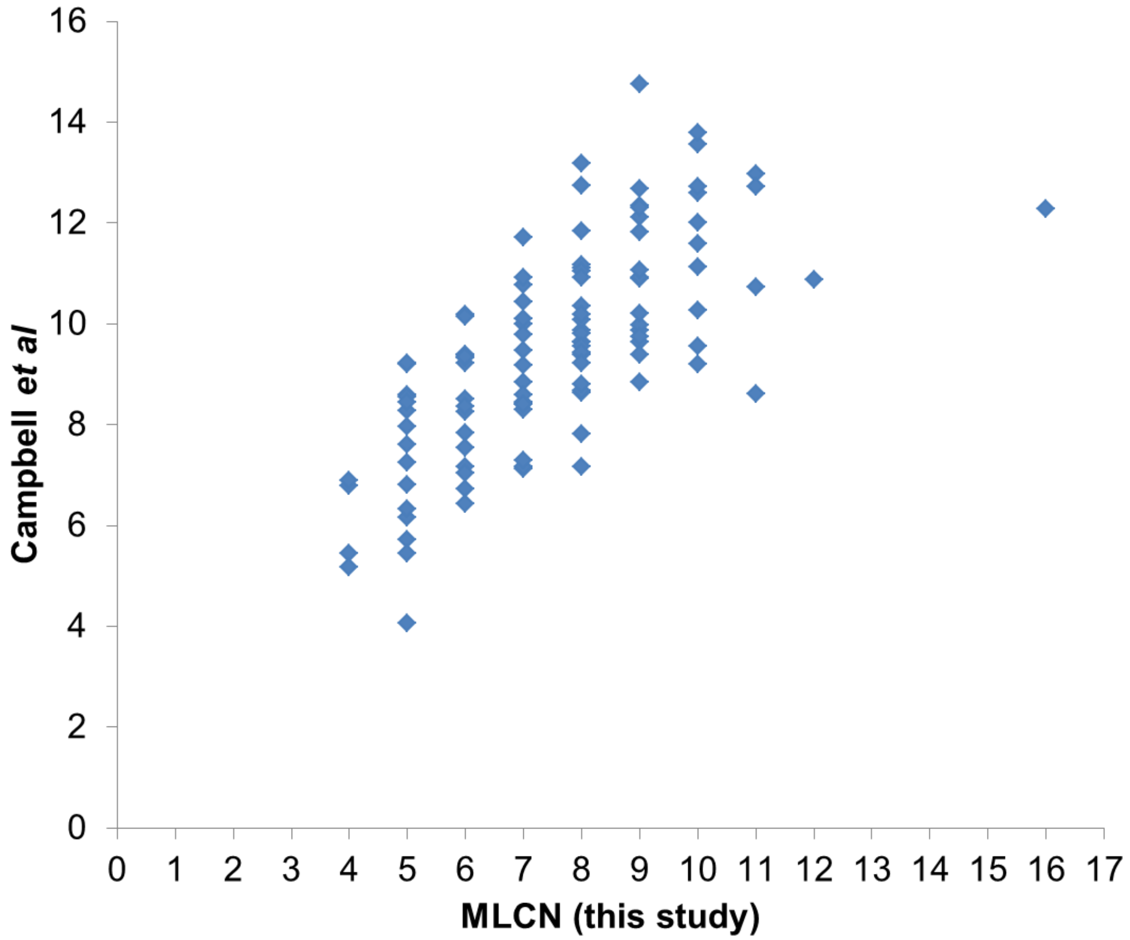

Supplementary Figure 5

Comparison between intensity data from Conrad et al. (2010) from the *DEFA1A3* CNV region for 17 samples and the MLCN from this study (see section 2 for details). The r^2 value is 0.74.

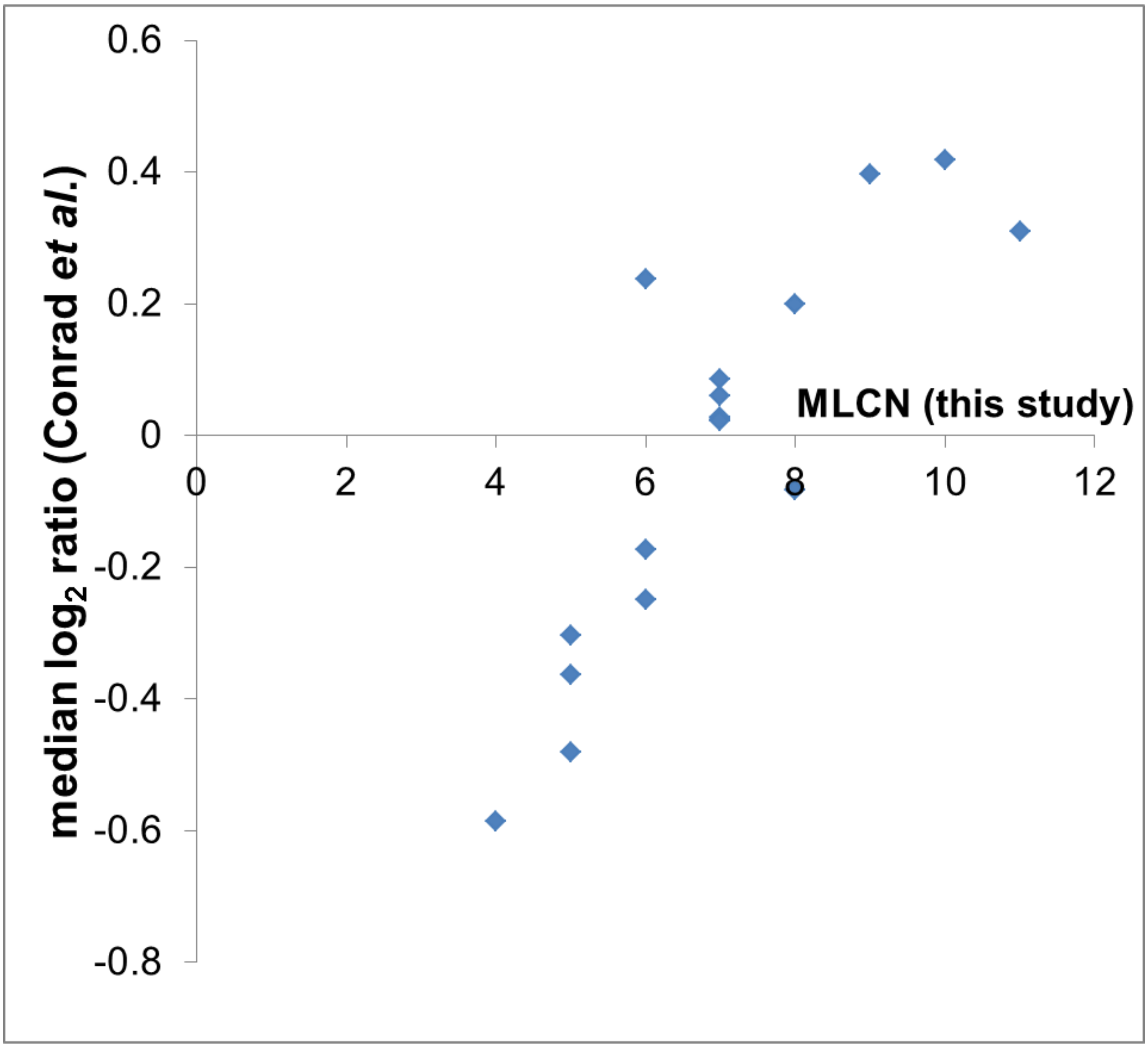

Supplementary Figure 6

Q-Q plot of all 87 non-duplicate P values for rs7825750, rs4300027 or rs4512398 from GWAS Central. To avoid double-counting of non-independent data, this compilation removed duplicate analyses of the same SNP in the same data, in every case choosing the more significant P value. This over-conservative procedure is likely to have led to the observed general small excess of non-significant P values in the range above P = 0.05 (i.e., $-\log_{10}$ P values below 1.3). Nevertheless, there is no indication of an excess of significant P values, and the overall pattern of P values observed is consistent with what would be expected from 87 numbers distributed randomly between 0 and 1.

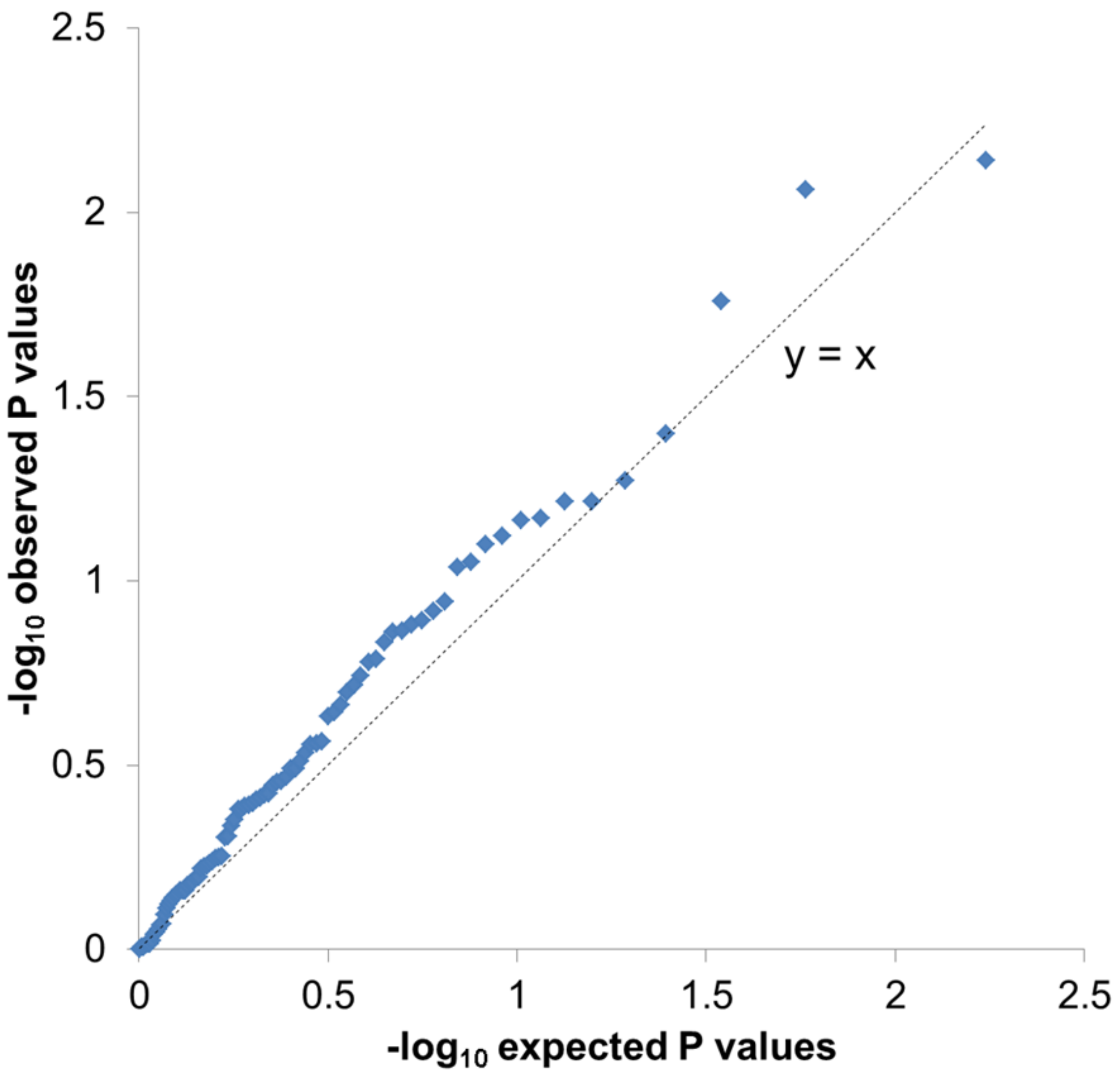

References (Additional File 1)

1. Aldhous MC, Abu Bakar S, Prescott NJ, Palla R, Soo K, Mansfield JC, Mathew CG, Satsangi J, Armour JAL: **Measurement methods and accuracy in copy number variation: failure to replicate associations of β-defensin copy number with Crohn’s disease**. *Hum Mol Genet* 2010, **19**:4930-4938.
2. Aldred PMR, Hollox EJ, Armour JAL: **Copy number polymorphism and expression level variation of the human alpha-defensin genes DEFA1 and DEFA3**. *Hum Mol Genet* 2005, **14**(14):2045-2052.
3. Khan FF: **Characterization of the Alpha Defensin Copy Number Variation in Humans**. *PhD thesis.* University of Nottingham, School of Biology; 2012.
4. Drmanac R, Sparks AB, Callow MJ, Halpern AL, Burns NL, Kermani BG, Carnevali P, Nazarenko I, Nilsen GB, Yeung G *et al*: **Human Genome Sequencing Using Unchained Base Reads on Self-Assembling DNA Nanoarrays**. *Science* 2010, **327**(5961):78-81.
5. Wellcome Trust Case Control Consortium: **Genome-wide association study of 14,000 cases of seven common diseases and 3,000 shared controls.** *Nature* 2007, **447**:661-678.
6. Dubois PCA, Trynka G, Franke L, Hunt KA, Romanos J, Curtotti A, Zhernakova A, Heap GAR, Adany R, Aromaa A *et al*: **Multiple common variants for celiac disease influencing immune gene expression**. *Nat Genet* 2010, **42**(4):295-302.
7. Hakonarson H, Grant SFA, Bradfield JP, Marchand L, Kim CE, Glessner JT, Grabs R, Casalunovo T, Taback SP, Frackelton EC *et al*: **A genome-wide association study identifies KIAA0350 as a type 1 diabetes gene**. *Nature* 2007, **448**(7153):591-594.
8. Silverberg MS, Cho JH, Rioux JD, McGovern DPB, Wu J, Annese V, Achkar J-P, Goyette P, Scott R, Xu W *et al*: **Ulcerative colitis-risk loci on chromosomes 1p36 and 12q15 found by genome-wide association study**. *Nat Genet* 2009, **41**(2):216-220.
9. Barrett JC, Hansoul S, Nicolae DL, Cho JH, Duerr RH, Rioux JD, Brant SR, Silverberg MS, Taylor KD, Barmada MM *et al*: **Genome-wide association defines more than 30 distinct susceptibility loci for Crohn's disease**. *Nat Genet* 2008, **40**(8):955-962.
10. Kugathasan S, Baldassano RN, Bradfield JP, Sleiman PMA, Imielinski M, Guthery SL, Cucchiara S, Kim CE, Frackelton EC, Annaiah K *et al*: **Loci on 20q13 and 21q22 are associated with pediatric-onset inflammatory bowel disease**. *Nat Genet* 2008, **40**(10):1211-1215.
11. Capon F, Bijlmakers M-J, Wolf N, Quaranta M, Huffmeier U, Allen M, Timms K, Abkevich V, Gutin A, Smith R *et al*: **Identification of ZNF313/RNF114 as a novel psoriasis susceptibility gene**. *Hum Mol Genet* 2008, **17** 1938-1945.
12. Liu Y, Helms C, Liao W, Zaba LC, Duan S, Gardner J, Wise C, Miner A, Malloy MJ, Pullinger CR *et al*: **A Genome-Wide Association Study of Psoriasis and Psoriatic Arthritis Identifies New Disease Loci**. *PLoS Genet* 2008, **4**(4):e1000041.
13. Esparza-Gordillo J, Weidinger S, Folster-Holst R, Bauerfeind A, Ruschendorf F, Patone G, Rohde K, Marenholz I, Schulz F, Kerscher T *et al*: **A common variant on chromosome 11q13 is associated with atopic dermatitis**. *Nat Genet* 2009, **41**(5):596-601.
14. International Multiple Sclerosis Genetics Consortium, Hafler DA, Compston A, Sawcer S, Lander ES, Daly MJ, De Jager PL, de Bakker PI, Gabriel SB, Mirel DB *et al*: **Risk alleles for multiple sclerosis identified by a genomewide study**. *The New England Journal of Medicine* 2007, **357**(9):851-862.

15. Fellay J, Ge D, Shianna KV, Colombo S, Ledergerber B, Cirulli ET, Urban TJ, Zhang K, Gumbs CE, Smith JP *et al*: **Common Genetic Variation and the Control of HIV-1 in Humans**. *PLoS Genet* 2009, **5**(12):e1000791.
16. Wright FA, Strug LJ, Doshi VK, Commander CW, Blackman SM, Sun L, Berthiaume Y, Cutler D, Cojocaru A, Collaco JM *et al*: **Genome-wide association and linkage identify modifier loci of lung disease severity in cystic fibrosis at 11p13 and 20q13.2**. *Nat Genet* 2011, **43**(6):539-546.
